# Investigating Infectious Organisms of Public Health Concern Associated with Wild Meat

**DOI:** 10.1155/2023/5901974

**Published:** 2023-08-18

**Authors:** Georgia Kate Moloney, Philippe Gaubert, Sophie Gryseels, Erik Verheyen, Anne-Lise Chaber

**Affiliations:** ^1^School of Animal and Veterinary Sciences, University of Adelaide, Roseworthy Campus, Adelaide, Australia; ^2^Global One Health Alliance Pty Ltd, West Lakes Shore, SA 5021, Australia; ^3^Laboratoire Evolution et Diversité Biologique (EDB), IRD/CNRS/UPS, University Toulouse III Paul Sabatier, Toulouse, France; ^4^Centro Interdisciplinar de Investigaçao Marinha e Ambiental (CIIMAR), University of Porto, Matosinhos, Portugal; ^5^Evolutionary Ecology Group, Department Biology, University of Antwerp, Antwerp, Belgium; ^6^OD Taxonomy and Phylogeny, Royal Belgian Institute of Natural Sciences, Brussels, Belgium

## Abstract

The wild meat trade poses a significant threat to public health as it facilitates the spillover of zoonotic pathogens through high-risk activities such as the hunting, butchering, trade, and consumption of wild animals. Despite the health risks and association with marking epidemics including SARS, Ebola, and COVID-19, the global wild meat trade continues to thrive. To summarize the evidence available, primary literature published between 2000 and 2022 was systematically and critically assessed for evidence of zoonotic pathogens or other infectious organisms detected in samples directly from wild meat, from animals hunted for wild meat, or from humans exposed through high-risk activities. Within the 97 articles analyzed, a total of 114 pathogen genera (15 viruses, 40 bacteria, 54 parasites, and 5 fungi) were detected in wild meat animals belonging to 168 vertebrate species including mammals, reptiles and birds sampled in 32 countries. In the context of wild meat specifically, infectious organisms were differentiated between those with zoonotic potential (32% of reported genera), ectoparasitic vectors (1%), and possible opportunistic or environmental contaminants. Thirteen viral, four bacterial, and one parasitic genera were also documented in humans participating in wild meat trade activities, supporting pathogen spillover potential. Most studies employed a targeted approach to evaluate the presence of (i.e., polymerase chain reaction (PCR); *n* = 65) or exposure to (i.e., ELISA; *n* = 19) a specific pathogen, while only one study employed broad-spectrum metabarcoding techniques. The diversity of infectious organisms associated with wild meat are highlighted through this review and could be used to guide policy development. However, the common use of a selected set of targeted detection assays likely biases the exploration of pathogen diversity, therefore potentially preventing the discovery of “disease x”. The global health risk demonstrated should make the illegal wild meat trade a priority for law-enforcement agencies and future research.

## 1. Introduction

Wildlife is trafficked locally and internationally on an immeasurable scale and is endangering public health. The wildlife trade involves the commerce of products, including meat, derived from non-domesticated animals, and therefore promotes close contact between humans and wildlife. Wild animals may act as reservoirs of infectious organisms, or be otherwise involved in the epidemiology of certain diseases. Over 70% of all emerging infectious diseases (EID) originate from an animal source [1, 2] and there is significant concern for “disease x”: a previously undescribed pathogen from wildlife that will initiate a pandemic in humans [3]. Epidemics including Severe Acute Respiratory Syndrome (SARS), Ebola, and Monkeypox (MPXV), as well as the recent COVID-19 pandemic (SARS-CoV-2), demonstrate disease emergence linked to the wildlife trade and wild meat consumption [4–7] and their substantial impact on the economy and human well-being [8, 9]. Therefore, it is vital to identify zoonotic and other infectious organisms of concern harbored by wild meat taxa and the high-risk practices which have contributed to, or have the potential to trigger, pathogen spillover and disease emergence events in humans.

For the purposes of this review, wild meat is defined as meat from a wild or non-domesticated animal. Wild meat traditionally serves as a reliable source of income and cheap source of nutrition for rural households, particularly in poorer regions, or as a result of leisure hunting [10]. The demand for wild meat is also fueled by its perceived health benefits or taste, where many consumers view it as a luxury product [11, 12]. The wild meat trade can be divided into the local trade, which involves the hunting and processing of meat for private sale or in marketplaces, and the international trade, which refers to the distribution of these products to consumers worldwide. While wildlife can be traded legally, illegal trafficking is thought to be very substantial, with estimates suggesting it generates USD $7–$23 billion annually [13]. The international wildlife trade is facilitated by its low prioritization as a serious crime and lack of effective law enforcement, despite significant costs to the economy, public health, and biodiversity. The complexity of the trade must be acknowledged, where each stage of the wild meat supply chain, including hunting, butchering, trade, and consumption, facilitates close contact between humans and wildlife and exposure to different risk factors [14, 15].

In this review we compile available research data on infectious organisms associated with wild meat and explore the link between human practices and risk of zoonoses connected to the local and international trade. The aim of our review was to summarize the laboratory findings from contemporary studies investigating infectious organisms associated with wild meat. We provide an overview of the scope of research currently being conducted in this field and collate a list of pathogens which have been identified in wild meat. Our review exclusively considers primary peer-reviewed literature and highly relevant doctoral theses demonstrating the presence of an infectious organism either from an animal hunted for wild meat, a sample of wild meat directly, or identified from a human involved in the wild meat supply chain with a highly suspected wild animal origin. The aim is not to quantify the level of risk, but to explore current pathogen surveillance capacity and identify research priorities. We discuss the likelihood of pathogen spillover events associated with different stages of the wild meat supply chain, comparing locally hunted and consumed wild meat with wild meat traded in marketplaces or transported internationally. Based on the findings of this review, suggestions are provided as to how infectious organism investigations in wild meat should be conducted in the future.

## 2. Materials and Methods

### 2.1. Search Strategy

A systematic literature review following the guidelines and procedures of the Preferred Reporting Items for Systematic Reviews and Meta-Analyses (PRISMA) [16] was conducted between 29 December 2021 and 8 January 2022. Three databases were selected to access a wide range of publications using the following Boolean search string: (“wild meat” OR wildmeat OR “bush meat” OR bushmeat) AND (zoono ^*∗*^ OR “zoonotic disease” OR “disease transmission” OR transmission OR sanitar ^*∗*^ OR pathogen ^*∗*^ OR disease ^*∗*^ OR “disease risk” OR “public health” OR health OR epidemic OR pandemic) AND (hunt ^*∗*^ OR process ^*∗*^ OR handl ^*∗*^ OR consumption OR trade). We chose the terms “wild meat” and “bushmeat” to search for literature on pathogen detections in non-domesticated animals, acknowledging that this term is more often used to refer to wild meat from the tropics where increased wildlife trade activity has been documented [17], while “game” usually describes meat from the Northern Hemisphere and leisure hunting. Consequently, there is a larger focus on research conducted in tropical regions in this review, but this is warranted as a disproportionate number of EIDs have originated in tropical regions, areas higher in mammal biodiversity and those experiencing land use changes [1, 18, 19]. Additionally, a pantropical distribution has been described for the wild meat trade in vertebrates, where regions including South and Southeast Asia, South America, and sub-Saharan Africa are more frequently implicated in the local and international wild meat trade [17].

To be included in this review, an article must directly reference “wild meat” or “bushmeat” in direct association with laboratory testing for infectious organisms in sampled wild meat products and/or humans participating in high-risk activities. Through our approach we effectively excluded studies that detected pathogens in wildlife known to be targeted by the wild meat trade but were sampled after capturing them in their natural habitat. As such, we acknowledge that our review likely underestimates pathogen diversity in species involved in the wild meat trade. Filters were applied to ensure only research published between 2000 and 2022 was included. The time period was chosen to capture the most recent data available with the consideration that detection tools have changed significantly in the last 20 years and relevant publications before this date were limited [20, 21].

### 2.2. Study Selection

**Figure 1:**
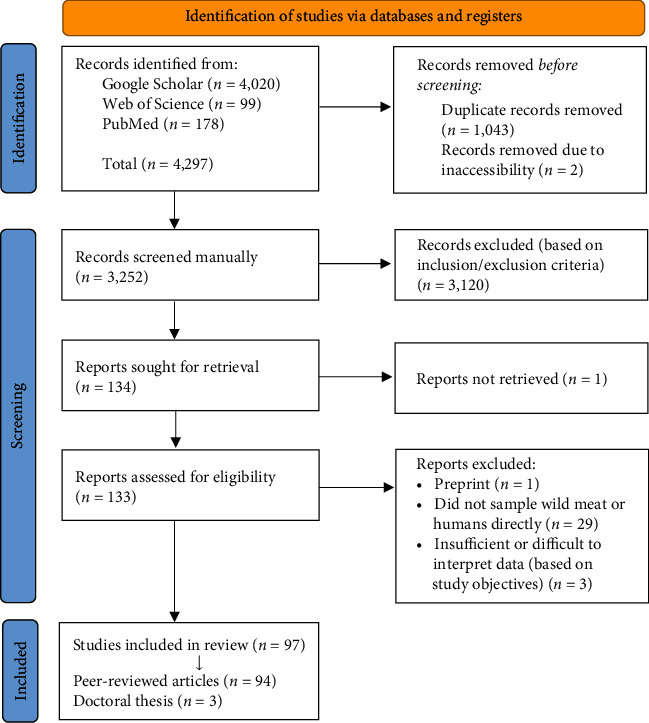
PRISMA flowchart illustrating the search strategy and systematic screening process for articles published between 2000 and 2022. The search criteria identified 4,297 articles, which was then refined as illustrated to produce 97 English language full-text articles for analysis.

Citations were imported into EndNote 20 (v20.2.1; [22]) and duplicates were removed. Only primary literature was selected wherein laboratory testing of infectious organisms occurred in samples of animals being hunted or processed as wild meat. Publications where samples from humans involved in the wild meat supply chain (i.e., hunting, butchering, handling, trade, or consumption of wild meat) were evaluated for the presence of zoonotic infectious organisms were also considered. In this instance, only papers demonstrating a strong link between the person's hunting activities or contact with wild meat and detected pathogen presence were included. One relevant paper was excluded as it was only available as a non-peer-reviewed preprint. Doctoral theses were also included as they are reviewed by a supervisory panel. All articles were assessed and deemed suitable for inclusion if they contained details regarding the presence of the infectious organisms screened for and found, information about the host, the screening methodology used and the country the samples originated from and were tested in. Papers were not excluded based on the sample types or methods used for pathogen screening. Articles which exclusively referred to risks associated with commercially farmed or non-terrestrial animals including pet or captive exotic animals (unless recently sourced from a wild meat vendor), or fish were excluded as they did not specifically relate to wild meat. A total of 97 articles were deemed suitable for inclusion based on the appointed selection criteria, including 94 articles from peer-reviewed journals and three doctoral theses. The overall search and inclusion process is presented using an adaptation of a PRISMA extension for scoping review flow diagram (Figure 1) [23].

### 2.3. Data Extraction

The parameters recorded and subsequently used for analysis were publication reference data, year of sample collection and/or analysis, animal species analyzed, if samples were directly derived from wild meat or instead from humans, sample type, sample size, positive sample size, detection method, category (whether infectious organisms screened were classified as viral, bacterial, parasitic, or fungal), and the continental region or country in which the study was conducted (including origin and destination for exported samples). Specific information regarding species sampled and pathogens detected were extracted and tabulated in Microsoft Excel [24], categorized by animal species and pathogen type (Supporting Information). Virus pathogen genera identification was based on the International Committee on Taxonomy of Viruses (ICTV) [25] while the bacteria genera was based on the International Committee on Systematics of Prokaryotes (ICSP) [26]. The organisms classified as parasites here refers to nematodes, platyhelminthes, protozoa, and ectoparasitic arthropoda. Only animal samples in which a potential infectious organism was identified were included. Studies wherein infectious organisms were either not tested for, reported or found were not included within the meta-analysis as they were not considered to be a proof of absence due to study limitations including restricted sample size, screening tool sensitivity, dubious sample quality, and inappropriate timing or sample type for the assessment of particular pathogens. Pathogen species-level identification was taken into consideration when categorizing infectious organism genera as zoonotic, if reported. Data analysis and visualization was conducted in Microsoft Excel [24] and R Studio (v3.3.0) [27] using the packages tidyr (v1.3.1) [28], dplyr (v1.0.6) [29] and ggplot2 (v3.3.6) [30].

## 3. Results

### 3.1. Infectious Organism Surveillance Methodologies

Methodologies employed for viral and bacterial pathogen detection varied between studies, wherein popular techniques included ELISA (*n* = 19) and western blot (*n* = 17), or a combination thereof. Polymerase chain reaction (including PCR variations) was described in 65 publications. In 49 studies, the PCR products were not further sequenced to allow for genetic characterisation of the strain. In 15 studies, positive PCR products were sequenced via Sanger sequencing, allowing the identification of a single organism per test. PCR products were sequenced using a next-generation sequencing approach (metabarcoding) in only one paper, allowing multiple bacteria strains present in the same sample to be identified. Methodologies specific to parasite detection included gross identification at the necropsy (three studies), faecal floatation (four studies), and sedimentation techniques (four studies).

### 3.2. Study Distribution

**Figure 2:**
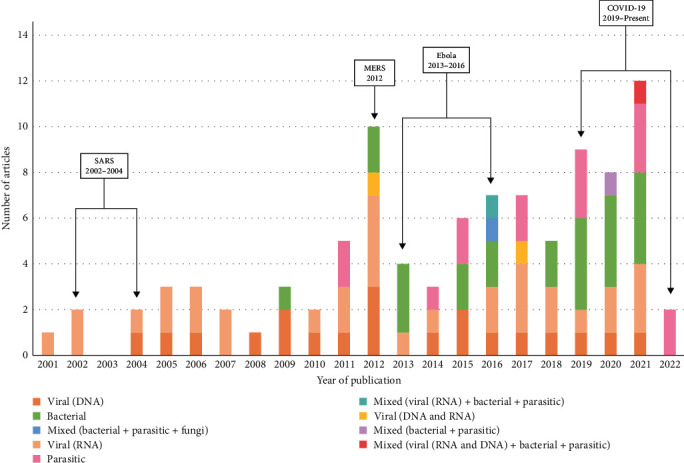
Distribution of peer-reviewed articles and doctoral theses published between 2000 and 2022 selected for review based on pre-determined inclusion and exclusion criteria. Each year is divided into the number of papers published per infectious organism type assessed (viral, bacterial, parasitic, or a combination of pathogens). The dates of key epidemic and pandemic events have been included above for reference.

**Figure 3:**
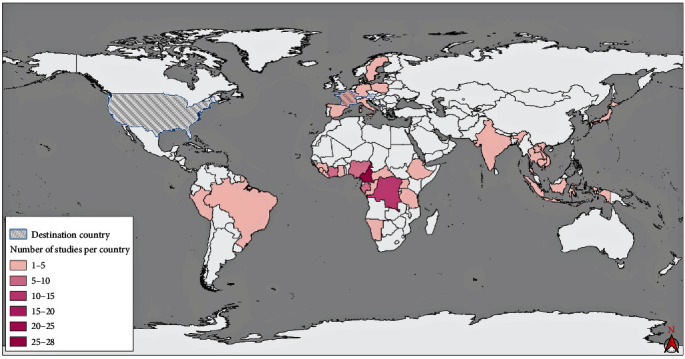
Global distribution of research papers included within the review. The color gradient represents the number of studies which were either conducted in or obtained samples from those countries (key provided). Nine studies involved more than one country and have been counted for each. Studies where samples originated from another country but were tested on importation are highlighted, being Austria (*n* = 1), France (*n* = 2), and the United States of America (*n* = 1). We used QGIS 3.24 to develop the map [31], with free data sets for country boundaries and land borders from “Natural Earth” [32].

There was a clear increase in the number of papers generated over time (Figure 2), particularly during (MERS 2012) or corresponding with the end (Ebola 2013–2016; COVID-19 2019–present) of major viral epidemics (Figure 2). Samples investigated in the selected papers were sourced from 32 countries, with a significant proportion either originating from or demonstrating some connection to regions of Africa (*n* = 83), particularly Central Africa (*n* = 50) including Cameroon (*n* = 28) and the Democratic Republic of Congo (*n* = 13). In addition, three countries tested wild meat specimens that had been imported from elsewhere, being Austria (*n* = 1), France (*n* = 2), and the United States of America (*n* = 1). The studies investigating samples from all continents were represented in the dataset; Europe (*n* = 9), Asia (*n* = 6), South America (*n* = 6), North America (*n* = 2), and Oceania (*n* = 1) (Figure 3). Humans participating in high-risk activities (i.e., hunting, butchering, trade, and consumption) were also investigated in 28 studies, largely conducted in West and Central Africa.

### 3.3. Infectious Organism Detection across Wild Meat Host Taxa

A total of 168 animal species were reported as carrying positive samples for 114 pathogen genera (15 viruses, 40 bacteria, 54 parasites, and 5 fungi; Table 1). Many of the genera reported in wild meat samples have members which are known to be zoonotic (37/114, 32%) or have the potential to cause illness in humans. Antibodies for zoonotic pathogens were also identified in 3.7% (995/27217) of the high-risk humans studied. Mammals were the most frequently reported taxonomic class, represented by 43 identifiable species of Chiroptera, 39 Primates, 39 Artiodactyla, 18 Rodentia, eight Carnivora, five Lagomorpha, two Perissodactyla, two Pholidota, one Cingulata, one Eulipotyphla, one Tubulidentata, and one Proboscidea. In addition, infectious organisms were documented in six reptile and two bird species. Animal sample size per species in which a pathogen was identified varied considerably, ranging from 1 to 1,107.

**Figure 4:**
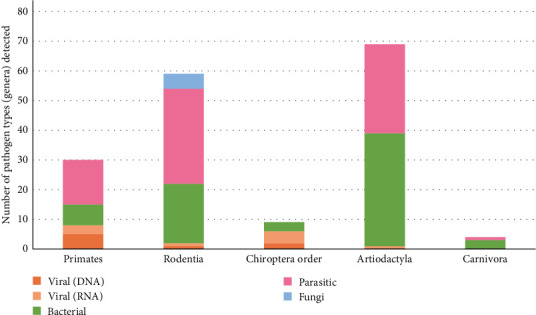
Pathogen identification in wild meat samples across the most frequently targeted and reported animal taxa (in relation to the number of pathogen genera identified). The columns represent the number and type of pathogens identified across all samples.

**Figure 5:**
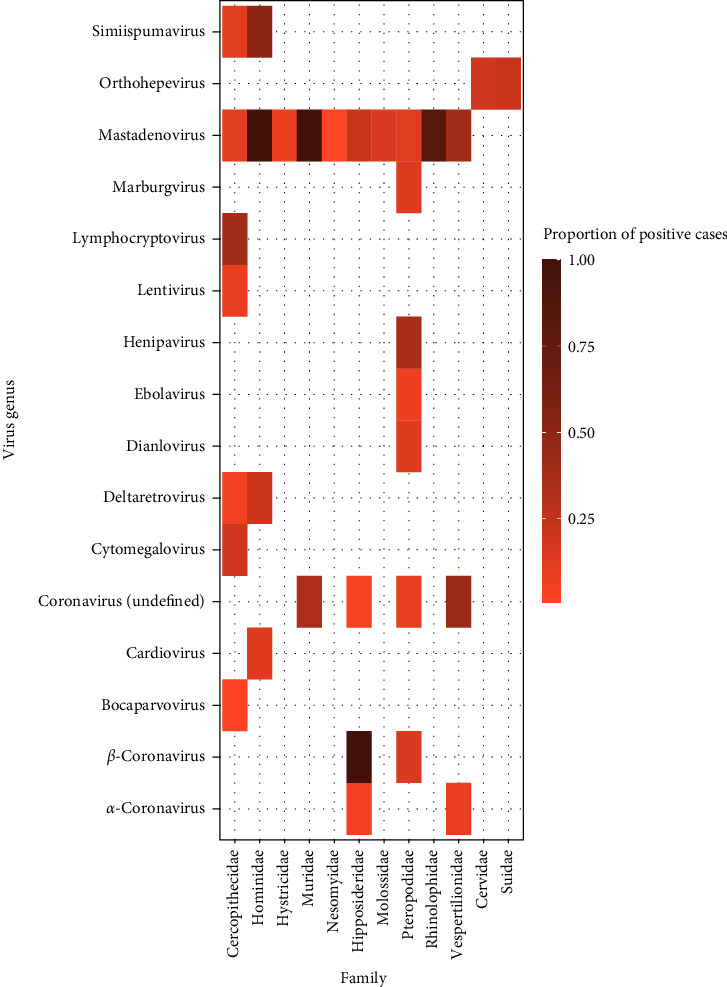
Heat map representing the proportion of positive cases reported for viral genera detected across wild meat samples of families within the orders Primates, Rodentia, Chiroptera, and Artiodactyla.

**Figure 6:**
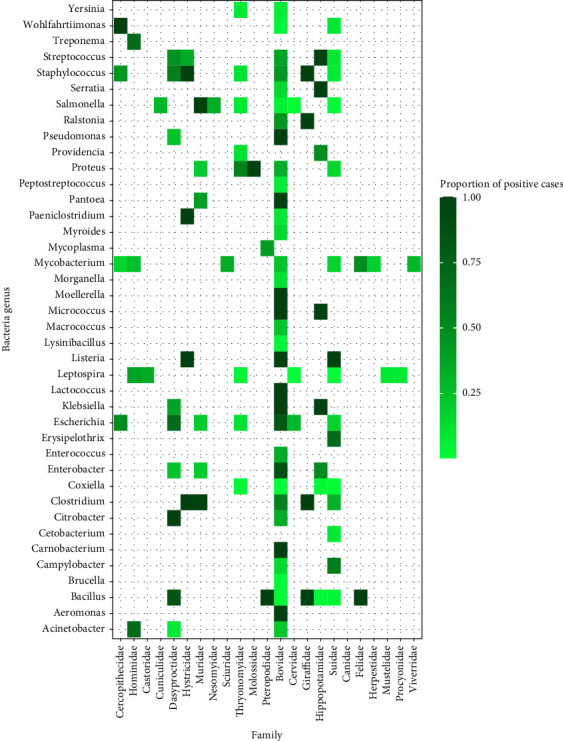
Heat map representing the proportion of positive cases reported for bacteria genera detected across wild meat samples of families within the orders Primates, Rodentia, Chiroptera, Artiodactyla, and Carnivora.

**Figure 7:**
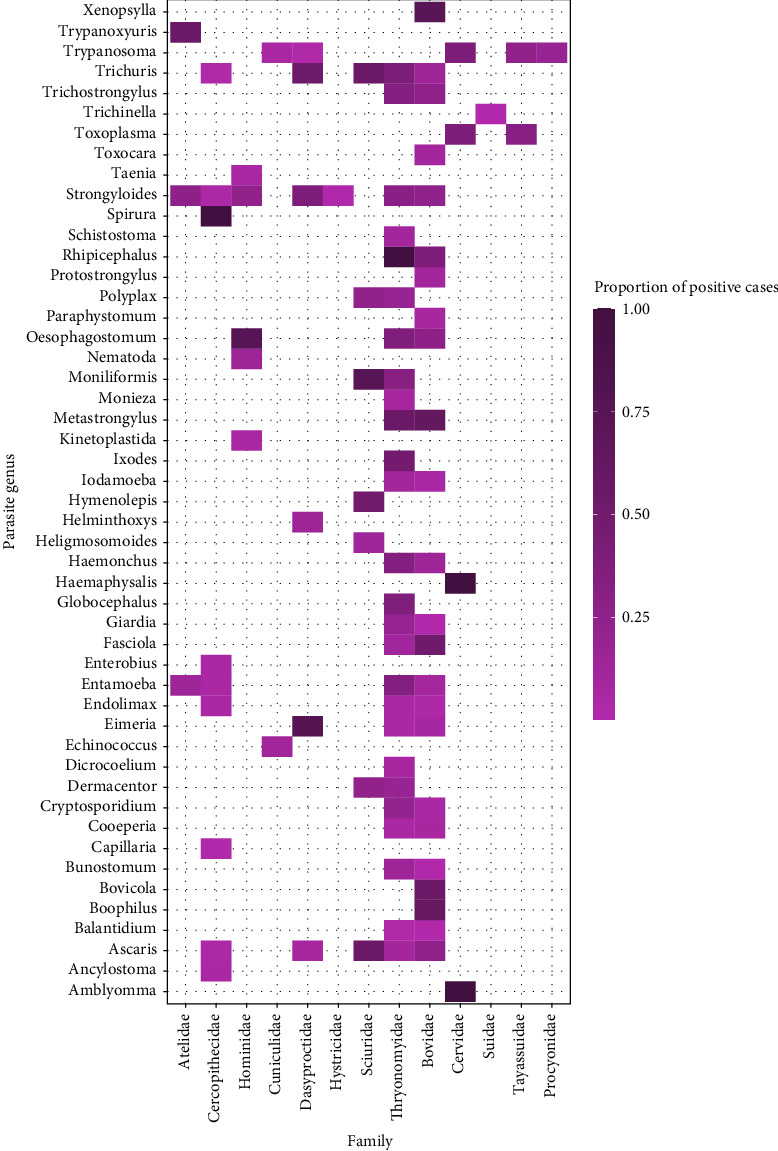
Heat map representing the proportion of positive cases reported for parasite genera detected across wild meat samples of families within the orders Primates, Rodentia, Chiroptera, Artiodactyla, and Carnivora. Cases of *Bertiella* and *Enterobius* in Cercopithecidae have been excluded as the number of positive samples was not disclosed. *Armillifer*, *Ascaridia*, *Dermanyssus*, *Goniocotes*, *Metadavainea*, and *Spilopsyllus* were not represented by these families.

There was variation in the types of pathogen genera detected across the most frequently targeted taxonomic orders (Figure 4). The number of different pathogen genera found was highest in Artiodactyla (*n* = 69), followed by Rodentia (*n* = 59), Primates (*n* = 30), Chiroptera (*n* = 9), and finally Carnivora (*n* = 4). Fungi were only reported in one study investigating rodents. As reporting frequency varied, the data was further deconstructed to represent the infectious organism genera detected in association with popular mammalian families (Figures 5–7). The detection frequency displayed for a given pathogen was determined as the number of positive wild meat animal samples divided by the total sample size for that species across the studies. However, as sample size, detection methodologies and host taxa investigated varied across studies, the summarized detection frequencies do not represent accurate prevalence numbers for the different pathogens.

### 3.4. Viruses

Across 56 studies, a total of 15 virus genera (RNA viruses *n* = 9 and DNA viruses *n* = 6) were reported to be detected in wild meat taxa. In 26 studies, evidence of transmission to humans was found. The most frequently reported virus genera in wild meat taxa were deltaretrovirus (10 studies) and lentivirus (10), followed by alpha- and betacoronaviruses (5), mastadenovirus (4), and simiispumavirus (2). Both the virus genera that were targeted in the laboratory assays and the actual detection frequency differed between the investigated mammalian families, mostly because the families investigated also differed across studies. Antibodies against 13 virus genera including deltaretrovirus, lentivirus, simiispumavirus, henipavirus, orthoherpesvirus, ebolavirus, and marburgvirus were also discovered in humans who reported direct contact with wild meat through hunting (including bites from non-human primates) and exposure to bodily fluids during meat preparation.

### 3.5. Bacteria

In 28 studies, wild meat taxa were associated with 40 bacteria genera, which were further discriminated between those with zoonotic species (30%) and those which are more likely to be environmental contaminants (68%, largely represented by *Enterobacterials*). Artiodactyls, particularly Bovidae, were associated with the greatest diversity of bacterial genera detected (38 genera over 10 studies), followed by Thryonomyidae (21 genera in 4 studies). Sample sizes varied considerably and therefore estimated prevalence could only be calculated in some instances. For example, in Bovidae the estimated prevalence of *Coxiella burnetii* (the causative agent of Q fever) was 1% [33], while *Salmonella spp*. were detected in 6% of samples [34, 35]. Three references provided evidence of human exposure to bacteria through direct contact with wild meat animals, where the zoonotic *Bacillus anthracis* (the causative agent for Anthrax) was reported in both wild meat species and humans butchering wild meat.

### 3.6. Parasites

We identified 54 parasite genera associated with wild meat animals across 19 studies, consisting of 42 endoparasites and 12 ectoparasites. Of these, 26% of the endoparasite genera (helminths and protozoa) reported across mammal and reptile specimens have zoonotic species, meanwhile only one ectoparasite (*Xenopsylla spp*.) was identified as a vector which could directly transmit zoonotic infections from wild meat species to humans. The greatest variety of parasites were found in Thryonomyidae (23 genera across 5 studies) and Bovidae (21 genera across 5 studies). Only one study investigated endoparasites in humans, wherein 8% were seropositive for *Echinococcus spp*., a parasitic tapeworm which can be obtained from either direct contact or consumption of contaminated meat.

## 4. Discussion

### 4.1. Research Priorities and Detection Tools

Publications studying wild meat increased from 2001 to 2021, in-line with the general increase in scientific publications per year [36], and subsequently demonstrated diverse infectious organism screening methodologies. The increased number of publications produced around recent epidemic and pandemic events suggests a retroactive approach to zoonotic disease research, meanwhile demonstrating a lack of proactive research (Figure 2). Similar to infectious disease investigations increasing retroactively with epidemics, it is likely that other studies followed key public health events or increased funding availability linked to awareness generated for the potential risks associated with wild animal hunting and trade [37]. The trend observed is also likely associated with the development of advanced screening tools such as metabarcoding for bacterial microbiome detection, wherein its use had increased by the end of the 2000s and become routine by the early 2010s [38]. PCR was utilized in a large proportion of studies (*n* = 65), which is a targeted approach as it amplifies a specific section of the genetic material. In most studies (*n* = 49), the PCR product was not further sequenced, therefore it remains uncertain which exact species or strain of virus, bacteria or parasite was detected beyond the general specificity of the PCR primers. Similarly, ELISA (*n* = 19) and western blot (*n* = 17) were widely used, however provide an indirect measure of previous exposure to an infectious agent rather than confirming the presence of the agent itself. As almost all reviewed studies used targeted laboratory assays, there is an obvious large underestimation of the diversity of infectious organisms present in wild meat. This was especially the case for viruses, which were always detected using pathogen-genus or pathogen-family specific tests. The choice of which laboratory test was employed and therefore which infectious organism was targeted mostly reflects pathogens recognized to have public health or economic consequences

Metagenomics offers a broader approach for pathogen investigation in samples without the need for gene-specific amplification [39, 40]. Similarly, metabarcoding allows for the generic screening of DNA viruses, bacteria and protozoa as, although amplicon based, it uses conserved primers that capture most of the microbial diversity [41], as demonstrated in one study published in 2019 [42]. Application of metagenomic sequencing techniques facilitates comprehensive unbiased pathogen identification within wild meat samples and minimizes the possibility of overlooking “disease x”. Pathogen detection protocols (targeted vs. non-targeted approach) and animal sample size per species varied considerably between the studies. Therefore, while a reliable diversity index or pathogen prevalence was impossible to be quantified for most species, detection frequencies for each infectious organism type across taxonomic families was calculated (Figures 5–7 and Supporting Information). The diversity of infectious organisms compiled may help to guide further studies where a pathogen of concern has been reported, or alternatively where data is seemingly deficient. Ideally, advanced screening techniques should be used for proactive disease surveillance to identify high-risk practises before pathogen spillover events occur.

### 4.2. Geographical Distribution and Surveillance Capabilities

Our review employed key search terms designed to capture research conducted in pantropical regions which have previously been identified as hot spots of wildlife trade activity [17] and EID events [1, 18, 19]. While hunting practices in the Northern Hemisphere and other regions were not excluded from this review, the terminology used may vary from “bushmeat” or “wild meat”. As a consequence, papers using alternative terms like “game” were not captured in our literature search, however there is not necessarily a lower risk associated with wild meat hunting in these regions. Majority of the research cited occurred in sub-Saharan Africa, meanwhile there was an evident lack of research from other regions prevalent in the wild meat trade including Southeast Asia, Latin America, and the Caribbean (Figure 3) [10], reflecting findings presented by Peros et al. [20]. Research deficiencies may be associated with a lower prioritization in these regions. Diagnostic molecular laboratories with pathogen testing and surveillance capabilities are also inadequate in many regions where wild meat is commonly consumed due to insufficient funding and resources [43, 44]. Even laboratory capacity in sub-Saharan and Central Africa, where the majority of wild meat data analyzed originated, is insufficient for the population size and rate of disease emergence [45, 46]. As the wild meat trade is primarily associated with more remote tropical regions that also have limited access to pathogen testing facilities, the surveillance of novel pathogens in the pre-emergence phase is challenged, delaying the implementation of protocols for the prevention of zoonotic spillover events.

### 4.3. Taxonomic Species Representation and Pathogen Detection in Wild Meat

Mammals were the most frequently studied class of vertebrates, followed by reptiles and birds. Mammalian species have been reported to dominate the wild meat trade [47] and are associated with a heightened public health concern due to a higher likelihood of cross-species pathogen transmission between phylogenetically related species [48]. The five most frequently studied orders were ungulates (Artiodactyla), rodents (Rodentia), primates, bats (Chiroptera), and carnivores, primarily represented by species of the families Bovidae (23 species), Muridae (seven species), Cercopithecidae (34 species), Pteropodidae (23 species), and Mustelidae (six species), respectively. According to Han et al. [49], these orders represent the most pathogen rich taxa particularly for zoonoses, with rodents being the most abundant zoonotic hosts [49, 50]. As infectious disease dynamics are shaped by host diversity [51], the accurate identification of species sold as part of the wild meat trade is crucial to forecast the risk of zoonotic spillover events. DNA-typing analysis has shown that misidentification rates of species sold and traded as wild meat could reach high proportions [52, 53]. Therefore, we suggest wild meat investigations are accompanied by standardized species identification procedures to ensure comparability and accuracy of survey methodologies and to better anticipate transmission risks.

### 4.4. Infectious Organism Diversity, Sanitary Risks and Zoonotic Transmission

Fifteen viral genera were reported in wild meat, wherein all except cytomegalovirus have members known to be associated with direct zoonotic transmission. One of the most frequently reported viral families was retroviridae, which was tested and found exclusively in non-human primate samples. Based on serological investigations, retroviruses were also the most frequently described viruses in wild meat handlers, all of whom reported exposure to non-human primate bodily fluids via hunting or butchering, with studies describing multiple seropositive cases of deltaretrovirus (human T-lymphotropic virus), lentivirus (human/simian immunodeficiency virus), and spumavirus (simian foamy virus) [54–68]. Other zoonotic viruses detected in both humans and wild meat animals (artiodactyls and bats) included orthohepevirus (hepatitis E), henipavirus, ebolavirus, and marburgvirus. The detection of these viruses at the human-wildlife interface raises serious concern of novel EIDs. Coronaviruses were investigated only in rodents and bats and were found in Muridae, Hipposideridae, Pteropodidae, and Vespertilionidae families, though typically in low proportions [5, 7, 69]. Prior knowledge and public health priorities likely influenced screening efforts directed toward particular host taxa, where for example primates are more likely to be the focus of viral screening efforts rather than artiodactyls. Furthermore, because we selected only studies in which the sampled animals were directly involved in wild meat handling or trade, the representation of animal taxa is skewed toward those targeted for consumption. Viruses reported in wild animals not captured for meat have not been represented, where for example coronaviruses were not reported in *Rhinolophus spp*. despite their role as a reservoir species for SARS-like coronaviruses [70, 71], likely due to the relatively small sample size of this taxon that can be gathered from the wild meat trade. As such, it is not possible to make a fair comparison of natural pathogen diversity between animal taxa based on the studies reviewed here. Nethertheless, the literature has demonstrated clear evidence of viruses with zoonotic potential in wild meat processed from non-human primates, bats, artiodactyls, and rodents which can transmit directly to humans through hunting, butchering, and consumption. We recommend that given the likelihood of spillover arising from these interactions, future studies should be aimed at pandemic prevention, including consideration of ecological and socioeconomic drivers. However, despite the significant public health risk posed, viral investigations are still not comprehensive or sufficiently resourced.

Of the 40 bacteria genera reported in wild meat samples, 30% have species which display zoonotic potential as they are known to be transmitted to humans via hunting, butchering or consumption. While various bacteria were detected across a broad range of mammalian species, most genera were detected in low proportions. Meanwhile, 68% of the bacteria genera reported were classified as environmental or opportunistic organisms which may not originate from or infect the animals, but have the potential to cause illness or disease in humans. For example, Chaber et al. [72] confirmed that while *Listeria spp.* was isolated in samples traded internationally, most of the bacterial flora cultured from imported wild meat were environmental and likely the result of contamination due to unhygienic handling. Similarly, bacteria of the order *Enterobacterials* which were widely reported across mammalian samples may result in human infections, but cannot be considered zoonotic. The often-clandestine nature of the trade promotes unhygienic practices, where there are no standards for wild meat processing, preparation, or preservation [73–75]. Wild meat practises therefore not only pose a health threat due to zoonoses, but also because of infectious organisms associated with improper handling, inappropriate meat preparation, and environmental contamination, which were also frequently reported in the literature.

A range of endoparasites (helminths and protozoa) were identified in fecal or blood samples of mammals and reptiles, where 26% of the genera have zoonotic species members that could be transmitted via wild meat practices. While some other endoparasites listed may infect humans, they require passage through an intermediate host or time to develop or sporulate in the environment before becoming infectious, hence they are highly unlikely to be transmitted directly from the animal during the butchering process. However, carcass contamination during handling or exposure to unsanitary market conditions can pose a severe health risk for this reason. For the zoonotic endoparasites reported, direct contact with hunted animals or the consumption of undercooked wild meat containing larval forms of helminths such as *Echinococcus spp*. reported in Cuniculidae [76] and *Trichinella spp*. in Suidae [77] can induce serious harm or even death. However, certain important endoparasites transmitted via meat such as *Sarcocystis spp*. and *Spirometra spp*. are not cited in the research, indicating future testing schemes should consider all common organisms known to be associated with meat [78]. Ectoparasites also feature a public health risk as they can act as important vectors of disease, however they are largely host-specific. Studies have hypothesized that hunters are at an increased risk of tick-borne diseases due to wild meat butchering or consumption [79, 80], though this is more likely the result of increased environmental exposure through hunting activities rather than direct transmission [81]. Only one study reported the presence of an ectoparasite of public health concern in a wild meat specimen [79]. The flea (*Xenopsylla spp*.) is an important ectoparasitic vector of zoonotic disease transmission as it could jump directly from a wild animal to a human and transmit diseases such as bubonic plague (*Yersinia pestis*) [82].

### 4.5. High-Risk Human Activities

Evidence of zoonotic transmission or pathogen spillover from wild meat animals to humans has been linked to high-risk behaviors promoting direct contact at the wildlife-human interface [83]. Antibodies against infectious organisms associated primarily with wild meat hunting and handling were identified in 3.7% of the humans studied [63, 84, 85], supporting the zoonotic spillover potential explored by Milbank and Vira [83]. Additionally, those that have not previously been reported as infecting humans could cause illness due to opportunistic transmission under unnatural conditions created by humans. The combination of ecosystem anthropisation, stress on endangered populations and close interactions with humans facilitated by the trade greatly increases the risk of zoonotic disease emergence [86]. Stress generated in live captured animals housed in cages prior to slaughter has also been shown to significantly increase the likelihood of pathogen spillover events [14, 50, 87, 88]. Consequently, the risk of zoonoses and EID events is heightened with the trade of live animals, whereas normally during hunting animals are immediately killed and the zoonotic pathogens they are potentially carrying stop replicating and eventually die themselves. Furthermore, multiple animals, often from different species and geographic origins, being brought together in close confinement in marketplaces further facilitates amplification and genetic recombination events and pathogen spread across host species, ultimately increasing the likelihood of spillover to humans [15, 50, 75]. Overall, the trade and related high-risk activities facilitate closer interactions between humans and wildlife and therefore provide multiple opportunities for infectious organism spillover events to occur.

### 4.6. Variation in Sample Quality

The comparability of detection data across studies provided a significant limitation, as sample size, type, and quality explored within the selected studies was diverse, where for the purposes of this review pathogen detection rates in fresh tissue samples were treated the same as in smoked meat, feces or blood. A large amount of wild meat is smoked, where smoked samples are less likely to contain detectable pathogens [89] and therefore may misrepresent the risks posed by handling wild meat before the smoking process, or alternatively mislead perceptions of risk where a product is incorrectly smoked [90]. A large quantity of wild meat is transported either smoked or dried in the international trade [91, 92], where these products can still carry infectious organisms of concern. The detection of pathogens including *Escherichia coli*, *Listeria spp*., *Salmonella spp*., simian foamy virus and herpesviruses have been demonstrated in smoked wild meat products in Africa and Europe [72, 73, 89, 90, 93, 94]. Retroviruses and herpesviruses have also been detected in smoked and dried non-human primate samples imported into the United States, hence enhanced surveillance methods are required to ensure the public health impact is minimized [94]. Sample quality and pathogen tropism should be considered in wild meat surveillance studies to ensure appropriate samples are obtained to enable all pathogens of concern to be detected.

### 4.7. Recommendations for Surveillance and Future Studies

It is likely that the pathogens identified are only the beginning and that the full list of potential risks associated with wild meat is yet to be uncovered. Hence, further research should aim to incorporate larger sample sizes and target wild meat taxa wherein infectious organisms have not been fully characterized. Enhanced wild meat surveillance protocols in high-risk regions is important to assess the likelihood of a pandemic event and to enable prevention at the local level before it becomes a global concern. The studies focused on the surveillance of zoonotic pathogens in imported wildlife products were also limited despite the extent of the international wild animal trade and reported evidence of infectious organisms [17, 72, 94]. One difficulty associated with wild meat disease investigations, particularly within viral studies, was that until recently the methodology employed was typically focused on testing for one or few specific pathogens and therefore may have missed other viral families or “disease x”. However, with the development of advanced tools such as metabarcoding and metagenomics, which enable the direct analysis of populations of genomes within a sample at once, screening capabilities of pathogens of public health concern will be enhanced [95]. A transdisciplinary approach which considers the risk of wild meat trade activities to animal and human population health alike is required to properly appraise pathogen diversity and dynamics and thus determine the possibility of an outbreak [96]. The Global Health Security Index revealed that no countries are fully prepared for future epidemic or pandemic threats, which is alarming considering recent outbreaks and evidence of zoonotic spillover events [83, 97].

## 5. Conclusion

The evidence assembled supports the presence of zoonotic and opportunistic or environmental infectious organisms which can be transmitted from wild meat to humans. The list of pathogens reported in wild meat compiled through this review provides valuable information regarding current research efforts and can be used to guide future investigations and policy development. Many of the pathogens identified are either zoonotic or have been implicated in the development of disease in humans, which should spark concern for public health authorities globally. However, most of the screening tools employed were target-based and may prevent the detection of “disease x”, meaning pathogens that could later emerge in humans as novel infectious diseases could go undetected. A transdisciplinary approach and standardized testing methodology are required to investigate and assess the impact of infectious organisms associated with wild meat. Despite the public health threat posed by the local and international wild meat trade, there are currently limited surveillance practices, legislative policies, and enforcement strategies in place. Therefore, policymakers and enforcement agencies have a responsibility to prioritize wild meat infectious organism surveillance efforts to enable early outbreak detection and prevent the next pandemic.

## Figures and Tables

**Table 1:** Complete list of pathogen genera identified in wild meat samples, or from animals derived from the wild meat chain.

Viruses (*n* = 56)		
^*∗*^*α*-coronavirus	^*∗*^Deltaretrovirus	^*∗*^Lymphocryptovirus
^*∗*^*β*-coronavirus	^*∗*^Dianlovirus	^*∗*^Marburgvirus
^*∗*^Bocaparvovirus	^*∗*^Ebolavirus	^*∗*^Mastadenovirus
^*∗*^Cardiovirus	^*∗*^Henipavirus	^*∗*^Orthohepevirus
Cytomegalovirus	^*∗*^Lentivirus	^*∗*^Simiispumavirus
Bacteria (*n* = 28)		
Acinetobacter	Klebsiella	Peptostreptococcus
Aeromonas	Lactococcus	Proteus
^*∗*^Bacillus	^*∗*^Leptospira	Providencia
^*∗*^Brucella	^*∗*^Listeria	Pseudomonas
^*∗*^Campylobacter	Lysinibacillus	Ralstonia
Carnobacterium	Macrococcus	^*∗*^Salmonella
Cetobacterium	Micrococcus	Serratia
Citrobacter	Moellerella	Staphylococcus
Clostridium	Morganella	Streptococcus
^*∗*^Coxiella	^*∗*^Mycobacterium	Treponema
Enterobacter	Mycoplasma	Wohlfahrtiimonas
^*∗*^Enterococcus	Myroides	^*∗*^Yersinia
^*∗*^Erysipelothrix	Paeniclostridium	
^*∗*^Escherichia	Pantoea	
Parasites (*n* = 19)		
*Endoparasites*		
Ancylostoma	^*∗*^Entamoeba	Oesophagostomum
^*∗*^Armillifer	Enterobius	Paraphystomum
Ascaridia	Fasciola	Protostrongylus
Ascaris	^*∗*^Giardia	Schistostoma
^*∗*^Balantidium	Globocephalus	Spirura
Bertiella	Haemonchus	^*∗*^Strongyloides
Bunostomum	Heligmosomoides	^*∗*^Taenia
Capillaria	Helminthoxys	^*∗*^Toxocara
Cooperia	Hymenolepis	^*∗*^Toxoplasma
^*∗*^Cryptosporidium	Iodamoeba	^*∗*^Trichinella
Dicrocoelium	Metadavainea	Trichostrongylus
^*∗*^Echinococcus	Metastrongylus	Trichuris
Eimeria	Monieza	Trypanosoma
Endolimax	Moniliformis	Trypanoxyuris
*Ectoparasites*		
Amblyomma	Dermanyssus	Polyplax
Boophilus	Goniocotes	Rhicephalus
Bovicola	Haemaphysalis	Spilopsyllus
Dermacentor	Ixodes	^Xenopsylla
Fungi (*n* = 1)		
Aspergillus	Mucor	Penicillium
Candida	Paecilomyces	

*Note.n* represents the number of studies investigating that pathogen type. The asterix ( ^*∗*^) indicates genera of which members are known to be zoonotic (including foodborne (meat) organisms), whereas the caret (^) indicates potential vectors (ectoparasites) which could transmit zoonotic pathogens directly from wild meat animals to humans.

## Data Availability

The data that support the findings of this study are openly available in “Exploring infectious organisms of public health concern associated with wild meat” at https://doi.org/10.17605/OSF.IO/KS9B4.
